# Kinome Profiling of Regulatory T Cells: A Closer Look into a Complex Intracellular Network

**DOI:** 10.1371/journal.pone.0149193

**Published:** 2016-02-16

**Authors:** Andrea Tuettenberg, Susanne A. Hahn, Johanna Mazur, Aslihan Gerhold-Ay, Jetse Scholma, Iris Marg, Alexander Ulges, Kazuki Satoh, Tobias Bopp, Jos Joore, Helmut Jonuleit

**Affiliations:** 1 Department of Dermatology, University Medical Center Mainz, Johannes Gutenberg-University, Mainz, Germany; 2 Institute of Medical Biostatistics, Epidemiology and Informatics (IMBEI), University Medical Center Mainz, Johannes Gutenberg-University, Mainz, Germany; 3 Institute for Immunology, University Medical Center Mainz, Johannes Gutenberg-University, Mainz, Germany; 4 Department of Developmental Bioengineering, University of Twente, Enschede, the Netherlands; 5 Pepscope BV, Utrecht, The Netherlands; Jackson Laboratory, UNITED STATES

## Abstract

Regulatory T cells (Treg) are essential for T cell homeostasis and maintenance of peripheral tolerance. They prevent activation of auto-reactive T effector cells (Teff) in the context of autoimmunity and allergy. Otherwise, Treg also inhibit effective immune responses against tumors. Besides a number of Treg-associated molecules such as Foxp3, CTLA-4 or GARP, known to play critical roles in Treg differentiation, activation and function, the involvement of additional regulatory elements is suggested. Herein, kinase activities seem to play an important role in Treg fine tuning. Nevertheless, our knowledge regarding the complex intracellular signaling pathways controlling phenotype and function of Treg is still limited and based on single kinase cascades so far. To gain a more comprehensive insight into the pathways determining Treg function we performed kinome profiling using a phosphorylation-based kinome array in human Treg at different activation stages compared to Teff. Here we have determined intriguing quantitative differences in both populations. Resting and activated Treg showed an altered pattern of CD28-dependent kinases as well as of those involved in cell cycle progression. Additionally, significant up-regulation of distinct kinases such as EGFR or CK2 in activated Treg but not in Teff not only resemble data we obtained in previous studies in the murine system but also suggest that those specific molecular activation patterns can be used for definition of the activation and functional state of human Treg. Taken together, detailed investigation of kinome profiles opens the possibility to identify novel molecular mechanisms for a better understanding of Treg biology but also for development of effective immunotherapies against unwanted T cell responses in allergy, autoimmunity and cancer.

## Introduction

Regulatory T cells (Treg) are essential master regulators of tolerance. Although there are several subsets of cells with regulatory capacity it is commonly accepted that CD4^+^CD25^+^Foxp3^+^ Treg are key participants in the orchestration of tolerance [1,2]. A number of immunological disorders comprising autoimmunity as well as tumor disease involve a perturbation in Treg numbers and/or functions [3]. The ability to monitor and manipulate Treg may improve diagnosis or therapeutic intervention for immunological disorders. To achieve this, sensitive and specific biomarkers are required and a deep and profound knowledge of the interaction of those markers is mandatory.

The suppressive mechanisms of Treg have been extensively studied [1,4,5]. Although Foxp3 induction and Foxp3-orchestrated expression of a number of Treg-specific molecules such as CD25, CTLA4, GITR and CD127 are thought to play a central role in Treg differentiation and function [6–8], several studies suggest the involvement of additional regulatory elements necessary for Treg phenotype and function [9,10]. Herein, signaling pathways and kinase activities play an important role in Treg biology. Nevertheless, our knowledge regarding intracellular signaling pathways controlling phenotype and function Treg subsets is still very limited and only single kinase activities were in the focus of research so far [10,11]. Additionally, Foxp3 is not a specific marker for human Treg alone as it is also expressed in activated Teff [12]. All prior observations concerning Foxp3 expression in Treg as well as in activated Teff suggest that more than Foxp3 is necessary for fully explaining the regulatory phenotype.

Thus, detailed analysis of transcriptional regulation as well as regulation of protein expression in cell populations is an important approach in order to identify molecules that are functionally relevant in different T cell subsets. Herein, a broad variety of techniques has been generated for expression profiling of mRNA transcripts, proteins of different cell compartments or for studying kinase activity. Each method covers a distinct range for analysis of either molecule expression or activation and therefore bares its own advantages and limitations due to the restricted analysis [13–15].

In order to obtain more insight into this Treg specific network and to reveal novel potential target molecules we assessed changes in the activation of cellular kinases using a kinome array. Phosphorylation of proteins on tyrosine, serine, or threonine residues by kinases is one of the key biochemical mechanisms of signal transduction. Knowledge of kinase activation is important not only for understanding normal cell behavior, but also for elucidating pathogenesis of diseases. So far, in most studies, only single kinase activities were analyzed in distinct T cell subpopulations [16,17]. Nevertheless, as signaling pathways display a complex network of interacting kinases, the aim of the present study was to address the multiple pathways governing human Treg function in a more detailed and comprehensive way at different activation stages using kinome profiling. In the past, this technique has been successfully applied for identification of novel pathways in peripheral blood mononuclear cells, T lymphocytes, and adipocytes or osteoblasts, as well as in hypertensive renal damage [18–23].

Analysis of Treg early after stimulation using a statistical linear model revealed down regulation of kinases involved in cell cycle progression, T cell activation and cytoskeletal reorganization. In parallel, several kinases such as EGFR or CK2, not yet described in the context of human Treg, were activated in Treg early after TCR stimulation. Importantly, the same analysis was performed in Teff. This allowed us to study not only up-regulation of kinase activity comparing resting versus activated Treg but also to compare signaling network between both T cell populations and thus to define the Treg-specific kinome that participates in Treg-specific phenotype and function.

## Material and Methods

### Isolation and stimulation of human T cell populations

**Fig 1:**
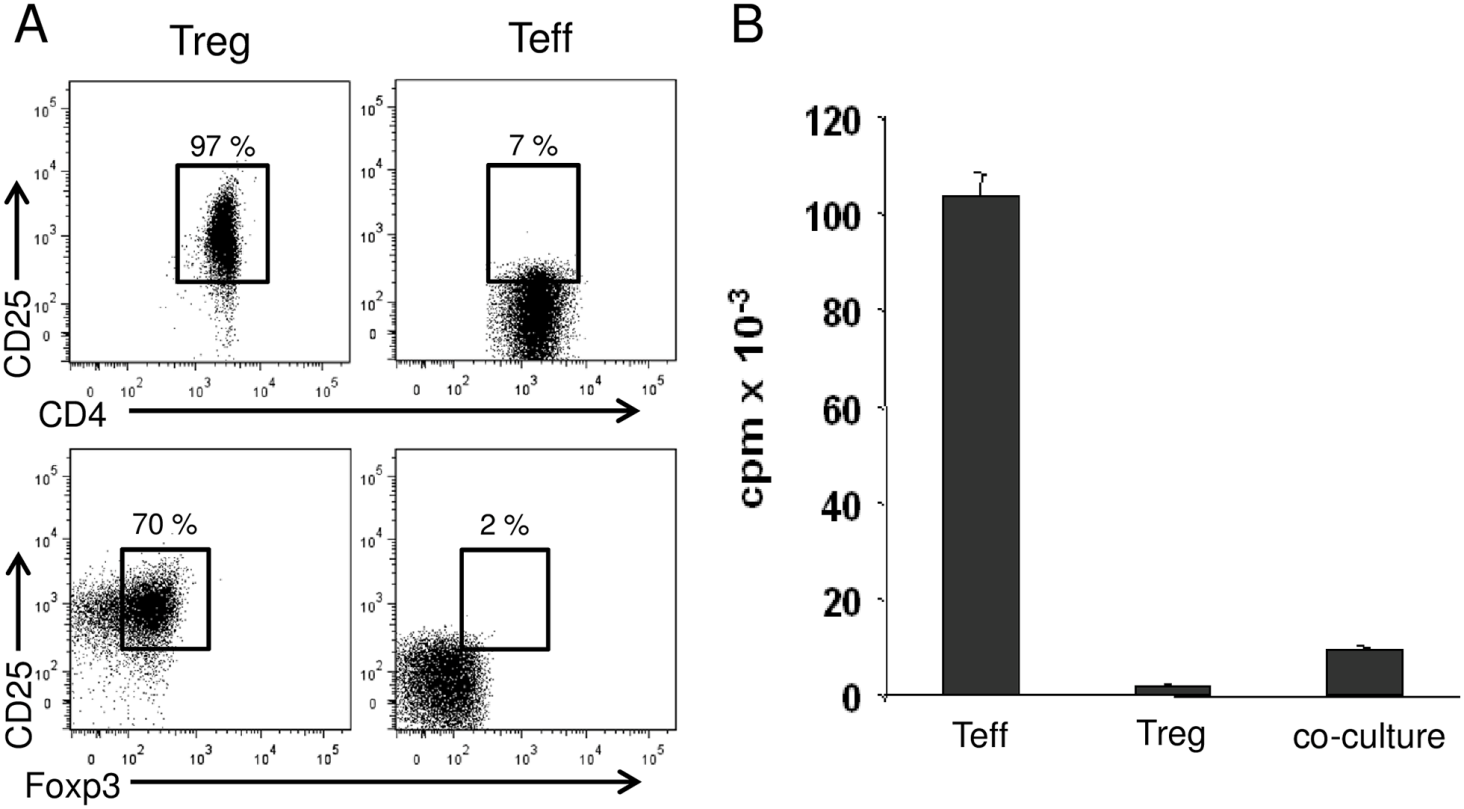
Characterization of human Treg subjected to kinome profiling. (A) After isolation, Treg were analyzed by flow cytometry regarding purity and phenotype. (B) Treg showed a characteristic suppressive function when co-cultured with Teff.

CD4^+^ T effector cells (Teff) and CD25^+^ Treg were isolated from leukapheresis products (up to 1.5 x 10^10^ whole cells) of adult healthy volunteers after informed written consent with approval by the local ethical committee (local ethics committee from Rhineland-Palatinate, Germany, No 837.029.05 (4687)) as described before [1,24]. Briefly, CD25^+^ cells were separated using limited amounts of CD25-microbeads (Miltenyi Biotec, Bergisch-Gladbach, Germany) resulting in CD25^high^ cells. Afterwards, contaminations of CD4^−^ cells were depleted using CD14-, CD8-, and CD19-Dynabeads (Invitrogen/Dynal, Oslo, Norway), resulting in a purity of CD4^+^CD25^high^ Treg greater than 95% (Fig 1A). CD4^+^ T cells were isolated using CD4-microbeads; afterwards, CD25^+^ T cells were depleted with CD25-Dynabeads (purity: CD4^+^CD25^-^ T cells greater than 98%). For polyclonal activation of T cells, 1 μg/ml anti-CD3 (OKT-3) and 2 μg/ml anti-CD28 (CD28.2; BD Pharmingen, San Diego, CA) mAb were used. In some experiments cells were labeled with carboxyfluoroscein succinimidyl ester (CFSE) or cell proliferation dye eFluor670 (CPD670; eBioscience) as indicated.

### Flow cytometry

Flow cytometric analysis was performed using the following antibodies: Mouse IgG: anti-CD25 (M-A25, BD Biosciences), anti-CD4 (RPA-T4, BD Biosciences). For intracellular staining of Foxp3, cells were fixed and permeabilized using a Fix/Permeabilization kit (eBioscience) and stained with anti-Foxp3 mAb (anti-human Foxp3-PE, clone 259D/C7, BD Biosciences). EGFR was stained using biotinylated EGF according to the manufacturer’s instructions (R&D Systems). Flow cytometry was performed on LSRII and Accuri C6 (BD Biosciences), data were analyzed using FlowJo software (Tree star).

### Peptide Array Analysis

Kinome array analysis was done as described by Diks et al. [25] and Löwenberg et al. [26]. Briefly, for kinome array samples, Treg and Teff were isolated and stimulated as described. Stimulations were terminated by an ice-cold phosphate-buffered saline wash. Cells were lysed in 200 μl of cell lysis buffer (20 mM Tris-HCl, pH 7.5, 150 mm NaCl, 1 mm Na_2_EDTA, 1 mm EGTA, 1% Triton X-100, 2.5 mm sodium pyrophosphate (phosphatase inhibitor), 1 mm MgCl_2_, 1 mm β-glycerophosphate (phosphatase inhibitor), 1 mm Na_3_VO_4_ (phosphatase inhibitor), 1 mm NaF, 1 μg/ml leupeptin (protease inhibitor), 1 μg/ml aprotinin (trypsin inhibitor), 1 mm PMSF (protease inhibitor)) and the volume of the cell lysate was equalized with distilled H_2_O. The cell lysates were subsequently cleared on a 0.22-μm filter. Peptide array incubation mix was produced by adding 10 μl of filter-cleared activation mix (50% glycerol, 50 μm [γ-^33^P]ATP, 0.05% v/v Brij-35, 0.25 mg/ml bovine serum albumin, [γ-^33^P]ATP (1000 kBq)). Next, the peptide array mix was added onto the chip, and the chip was kept at 37°C in a humidified stove for 90 min. Subsequently the peptide array was washed twice with Tris-buffered saline with Tween, twice in 2 m NaCl, and twice in demineralized H_2_O and then air-dried. Results include 3 technical replicates on each array performed.

### Peptide array image analysis and preliminary statistical evaluation

A dedicated image analysis procedure was used for quality control and quantification of spot intensities (Matlab 2013a, Mathworks, Natick, MA). As a first step, each kinase substrate was located by optimally aligning the grid of spotted substrates. Spot intensities were corrected for local background intensity. Following 2log-transformation, net spot intensities were subsequently analyzed. Data from three technical replicates were exported to an excel sheet for further analysis. Control spots on the array were analyzed for validation of spot intensities between the different samples. Furthermore, inconsistent data (e.g. spots with irregular shape or position) were excluded from further analysis. Slides were normalized by median-centering and a t-test was used to detect significantly different phosphorylation levels between different conditions.

### RT-PCR and qRT-PCR

RNA was extracted from 10^6^ cells using Rneasy Kit (Qiagen) according to manufacturer's instructions. cDNA was generated by reverse transcription using Sensiscript RT Kit (Qiagen). Gene expression levels were determined by quantitative RT-PCR (Applied Biosystem) and the QuantiFAST PCR Kit (Qiagen). PAK2, EGFR, IRF-1, TGFβ-R and CK2 (QuantiTect Primer, Qiagen), were normalized to the housekeeping gene EF1α (forward: 5'-gattacagggacatctcaggctg-3', reverse: 5'-tatctcttctggctgtagggtgg-3'). Relative mRNA expression was calculated in reference to Teff samples for EGFR, IRF-1, TGFβ-R and CK2 using the delta-delta Ct method.

### Western blot analysis

Nuclear and cytoplasmic fractions were prepared using nuclear extraction kit (Active Motif) according to the manufacture’s instruction. Fractionated proteins were assessed using a BCA (bicinchoninic acid) kit (Pierce), separated on a 4–12% sodium dodecyl sulfate—polyacrylamide gel (Novex), and transferred to a polyvinylidene fluoride (PVDF) membrane (Invitrogen). Membrane was immersed for 1 h in blocking solution (5% milk powder (Roth) in PBS containing 0.1% Tween 20 (PBS-T)) and probed with a molecular specific antibody in blocking solution overnight at 4°C. The membranes were washed extensively in PBS-T and incubated with horseradish peroxidase (HRP)-conjugated secondary antibody in blocking solution at room temperature for 1h. Blots were washed and visualized with enhanced chemiluminescence (ECL; Amersham). Polyclonal antibodies against PAK2 were purchased from Santa Cruz. The Laminin A/C polyclonal antibody (Cell Signaling) and actin monoclonal antibody (Sigma) were used as loading controls for nuclear and cytoplasmic fractions, respectively.

### Suppression assay

After isolation, Treg and Teff were cultured either alone or in co-culture at different ratios as indicated and stimulated with anti-CD3 (1μg/ml) and anti-CD28 mAb (2μg/ml) or T cell-depleted PBMC. Where indicated, Treg were pre-cultured with the inhibitor peptide Ht31 or a control peptide (Ht31P 23aa, both from Biaffin Kassel, Germany) at a concentration of 10μg/ml. T cell proliferation was measured after 4 days of incubation and an additional 16h pulse with [^3^H] TdR (37 kBq/well) using a liquid beta-scintillation counter or by use proliferation dyes via flow cytometry. In some experiments EGF (5ng/ml or 20ng/ml), anti-EGFR mAb (Cetuximab; 1μg/ml) or EGFR kinase inhibitor (Gefitinib; 0.5μM) was added as indicated.

### Bioinformatics/Statistical analysis

Missing values: Missing values for the technical replicates were imputed with the mean of the available values. Substrates with more than one missing value were excluded from the further analysis. Thus, 850 kinase substrates were used in the analysis.

Analysis of the linear model: For finding kinases of interest, we used a linear model. We utilize the R package nlme (version 3.1–105). Time point (0 min, 5 min, 15 min and 60 min), cell type (effector and regulator T cells) and technical replicate (1, 2 and 3) are used as covariates in the model. Due to the large number of tests applied in this study p-values have to be interpreted with caution and in connection with effect estimates.

In addition, we calculated the false discovery rates (FDR) for the model parameters time, cell type and technical replicate based on the Benjamini-Hochberg procedure.

Furthermore, we compared for each kinase a null model to the previously defined linear model with ANOVA. For multiple testing corrections we used the Bonferroni method.

## Results

### Identification of Treg-specific kinases

So far, in most studies, only single kinases were analyzed in distinct T cell subpopulations using mass spectrometry as a read out system. Nevertheless, as signaling pathways display a complex network of interacting kinases and do not only depend on presence or absence of the kinase but on activity, we studied the kinome network in more detail with a special focus on kinase activity. For identification of kinases specific for human Treg in comparison to Teff, both populations (S1 Fig) [27] were subjected to kinome profiling in order to identify specific kinase activity. In detail, sufficient numbers of Treg and Teff were isolated from leukapheresis products of healthy volunteers with a yield of up to 120x10^6^ Treg and a purity of >95% as described before [28]. Treg showed typical surface marker expression as well as suppressive function (Fig 1A and 1B).

After isolation, a part of Treg and Teff were stimulated with anti-CD3 mAb and anti-CD28 mAb. Samples were collected directly after isolation (0h) as well as after 5min, 15min and 60min of stimulation, lysed and compared with respect to their capacity to phosphorylate peptide substrates on arrays. This procedure resulted in the specific incorporation of ^33^P in a variety of peptides distinct for the time point and the cell populations analyzed (S2 Fig). The resulting slides for all conditions were analyzed, a median centering to normalize between the slides was performed and a subset of unreliable spots (4–12% per slide) was discarded. On the remaining spots, a t-test was performed to identify differences between conditions.

The resulting investigation revealed fold changes of each single kinase and its substrates comparing Treg over Teff. Importantly, the simultaneous analysis in Teff allowed us to study not only up-regulation of kinase activity comparing resting versus activated Treg but also to compare signaling network between both T cell populations and thus to define the Treg-specific kinome that participates in Treg-specific phenotype and function.

**Fig 2:**
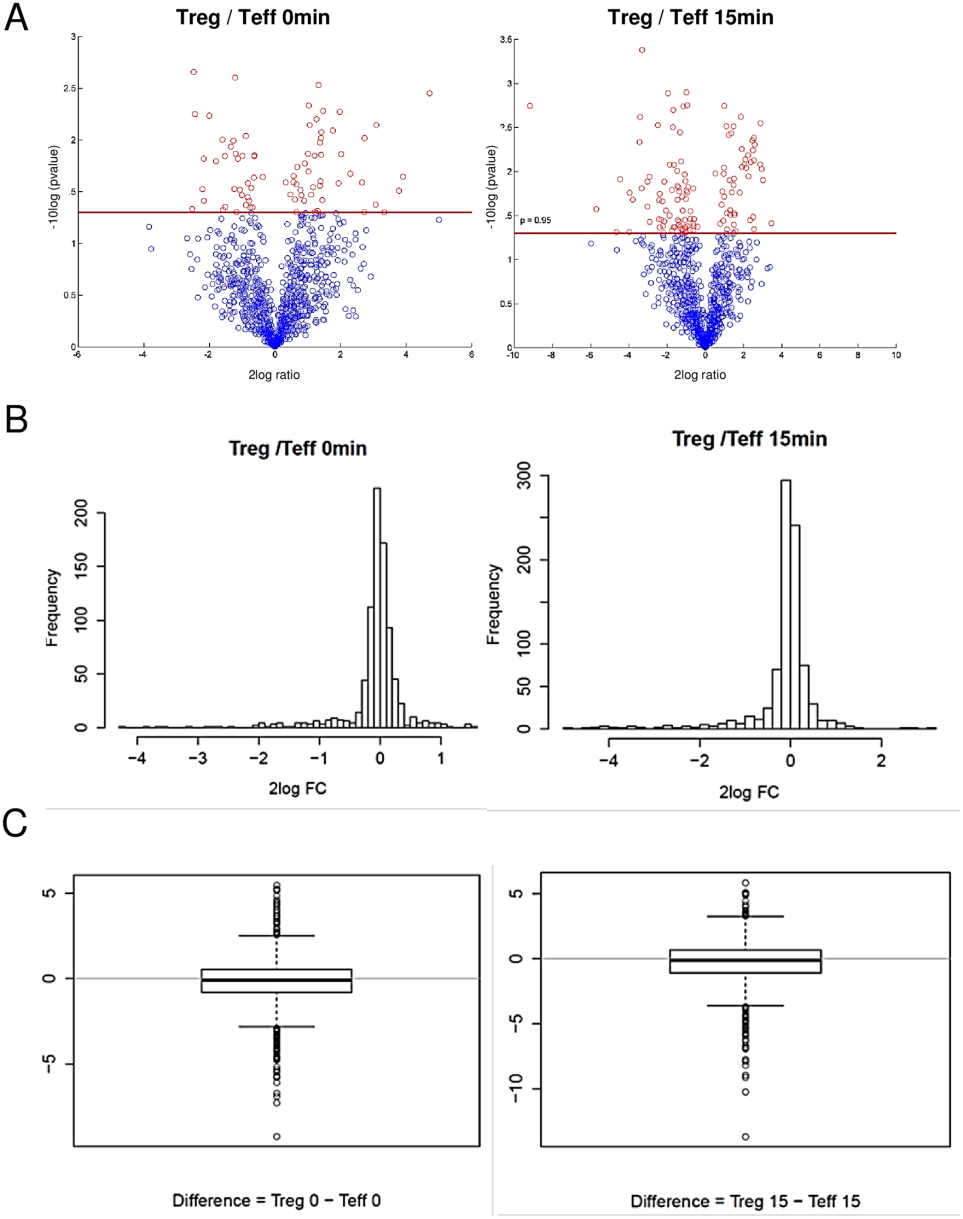
Distinct kinase activities in Treg compared to Teff. (A) Vulcano plots show the distribution of log ratios of all kinase consensus substrates from activated Treg versus activated Teff at different time points. (B) Histograms show the distribution of the frequencies of log2 FC for activated Treg and activated Teff at time point 0min (left panel) and 15min (right panel). (C) The differences between activated Treg and Teff at time points 0min and 15min are shown.

**Fig 3:**
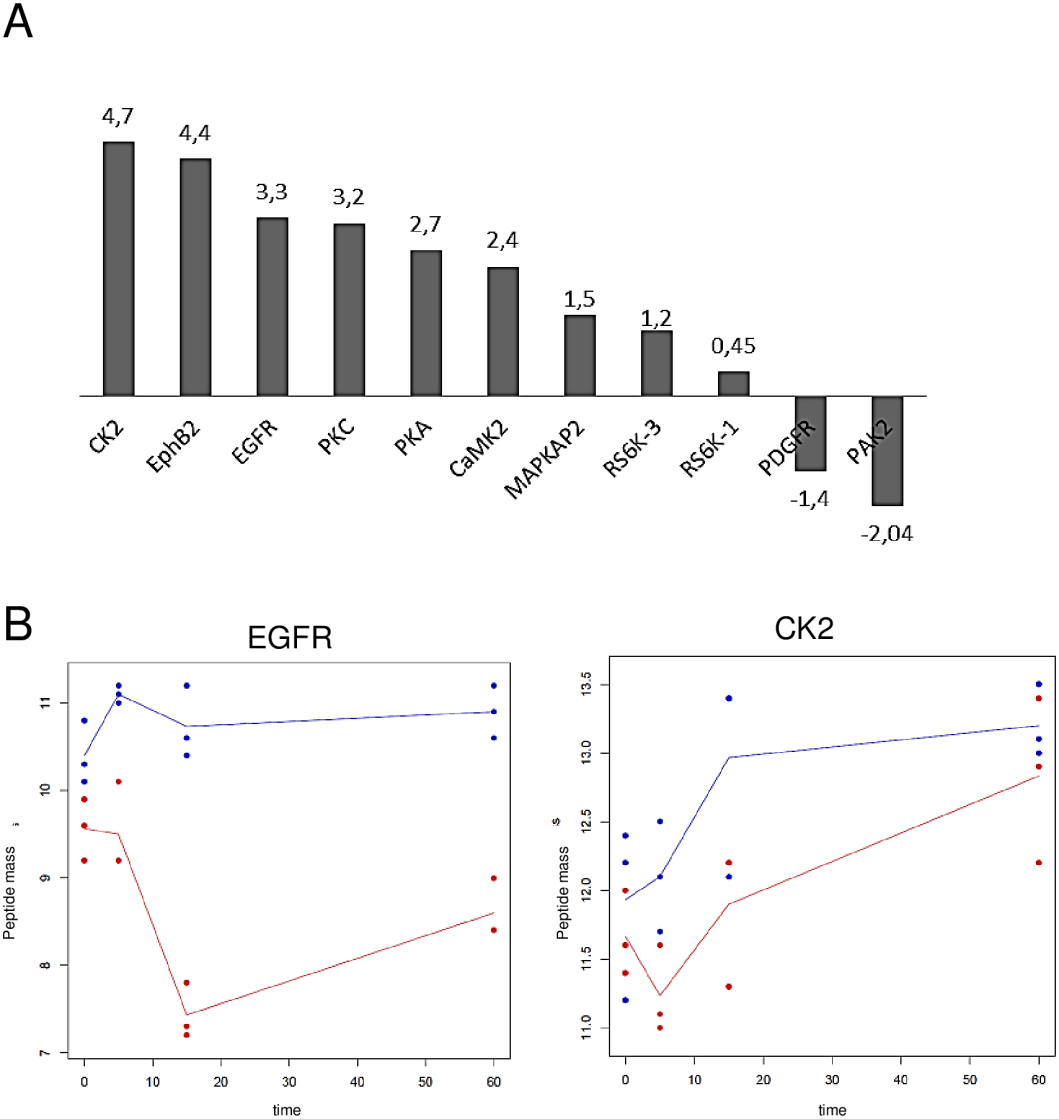
Distribution of kinase activities in Treg compared to Teff. (A) Bars indicate fold increase over control of upstream kinases that showed significant differences in regulation between Treg and Teff. (B) Graphical illustration of the kinetic profiles of the two peptides CK2 (right) and EGFR (left) for Treg (blue) and Teff (red). For every time point measured expression of the peptide for all three samples is depicted for both cell types as dots. The curves depict the mean expression for the peptides, separated for Treg and Teff, over all time points.

Already the comparison of resulting volcano plots gave us a first hint, that, in general, the majority of kinases was not exclusively expressed or differentially regulated in either Treg or Teff (Fig 2A). However, the comparison of volcano plots is a rather descriptive way to interpret the obtained data and thus resulting values were demonstrated additionally as histograms of the log2 FCs and boxplots of differences between Treg and Teff (Fig 2B and 2C). As a more advanced statistical tool for data interpretation we used a linear model for statistical analysis (see also Material and Methods). This model allowed us to adjust for the different time points, cell types and technical replicates present in the experiment. Using this approach we were able to obtain statistically more valid results compared to the descriptive t-test. Based on this linear model we found 11 kinases of interest which were regulated in a statistically different way when comparing Treg to Teff (Table 1, Fig 3A). Analysis of expression profiles of found kinases over the time illustrated that differences between differentially regulated kinases and their activation state achieved its maximum within the first 20 minutes after activation (Fig 3B for CK2 and EGFR, S3 Fig for other kinases).

**Table 1:** Identification of significant differences between kinome profiles in activated Treg versus activated Teff using a linear model.

Kinase	p-value	Substrate	Peptide	Phosphorylation site
CK2	3,52 x 10^−7^	Cell division cycle 34	APDEGSDLFYD	S203
CaMK2	6,95 x 10^−6^	C ets 1 protein	VPSYDSFDSED	S285
PKA	1,65 x 10^−5^	NFKB3	QLRRPSDRELS	S263
EGFR	1,99 x 10^−5^	Ezrin	LRLQDYEEKTK	Y307
PKC (alpha subunit)	2,05 x 10^−5^	c-Src	KPKDASQRRRS	S12
EphB2	4,21 x 10^−5^	Ras-related protein	TIEDSYTKIAS	Y66
PAK2	5,32 x 10^−5^	Ribosomal protein S6	RRRLSSLRAST	S236
MAPKAP kinase 2	9,99 x 10^−6^	Serum response factor	LKRSLSEMEIG	S103
Ribosomal S6 kinase 1	4,38 x 10^−5^	Protein phosphatase 1	RRGSDSSEDIY	S48
PDGFR beta	1,27 x 10^−7^	SHP2	RKGHEYTNIKY	T353
Ribosomal protein S6 kinase alpha 3	4,89 x 10^−5^	HMG14	EPKRRSARLSA	S7

For finding kinases of interest, we fit a linear model by using the R package nlme (version 3.1–105). Different time points (0 min, 5 min, 15 min and 60 min), cell types (Teff and Treg) and technical replicates (1, 2 and 3) were used as covariates. The table shows the results for different kinase regulation comparing Treg and Teff and the corresponding p-values. In addition, substrates including peptide sequence and phosphorylation site in which phosphorylation was significantly upregulated by their corresponding upstream kinase are presented. CaMK2, Calmodulin dependent kinase 2; EphB2, Ephrin B2; PKC, protein kinase C; MAPKAP 2, mitogen-activated protein kinase-activated protein-2; PKA, protein kinase A; PDGFR, platelet-derived growth factor receptor; EGFR, epidermal growth factor receptor; PAK2, p-21 activated kinase 2; CK2, casein kinase 2; Only kinases with significant differences are shown.

Taken together, we observed especially quantitative differences in both populations. Thus, resting Treg showed an overall lower number of activated kinases, the majority of kinases in Treg were down regulated compared to Teff (Fig 2A). Nevertheless, within 20 min after activation we detected a distinct pattern of activated kinases when compared activated Treg to activated Teff (Fig 3A and 3B, Table 1). Importantly, up- and down-regulation of kinases not yet discussed in the context of Treg were observed. As an example, Treg showed an altered pattern of CD28-dependent components and of kinases involved in cell cycle progression and cytoskeletal reorganization such as PAK2, also described as a positive regulator of T cell activation that interferes with NFAT expression and IL-2 production. Additionally, significant up-regulation of kinases in activated Treg but not in Teff such as EGFR or CK2 demonstrated that a specific molecular activation pattern defines the activation state of human Treg.

### Expression levels of selected kinases in human regulatory T cells

**Fig 4:**
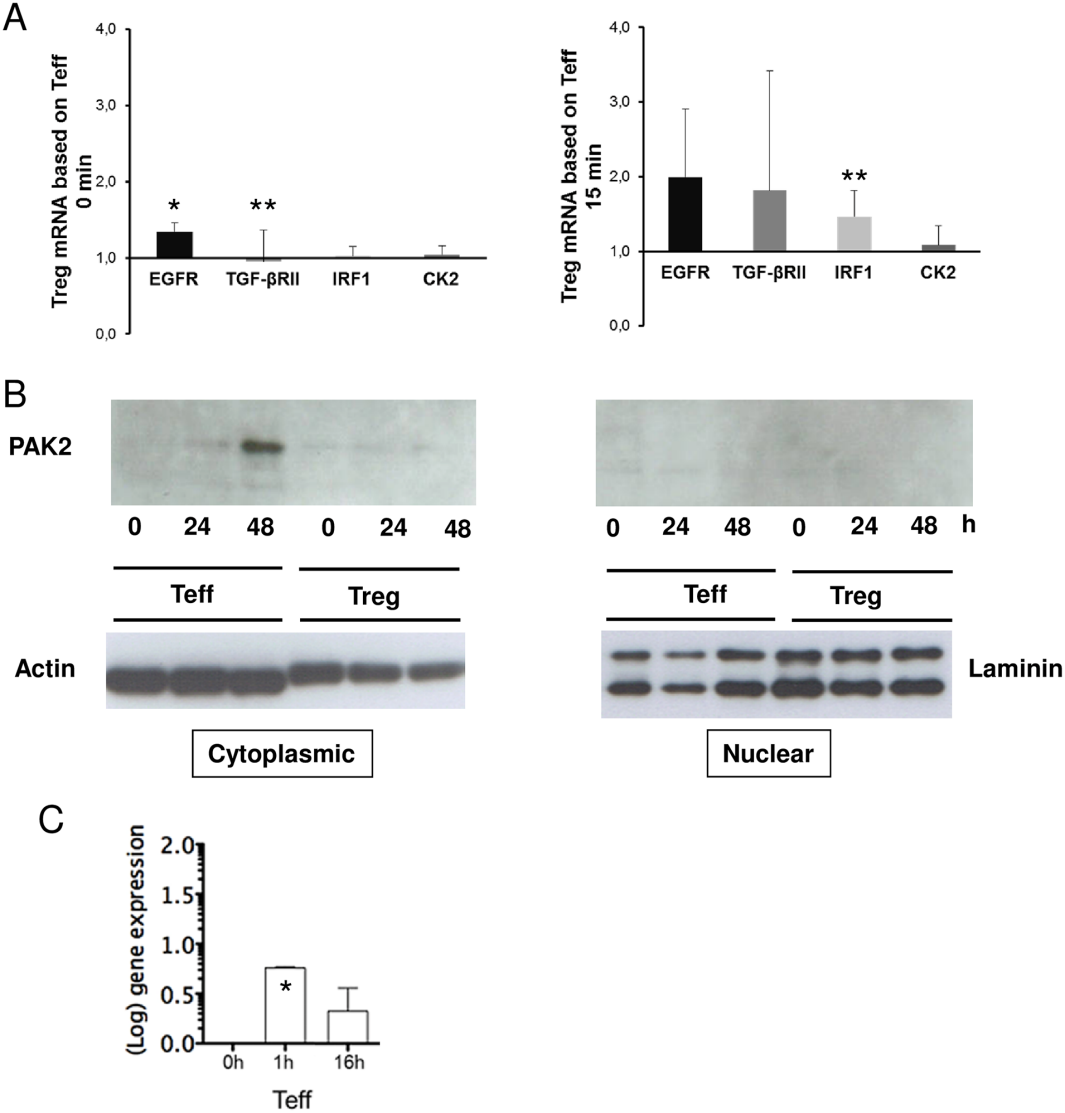
Expression levels of marker molecules via qRT-PCR and Western blot. (A) Treg or Teff were untreated or stimulated with anti-CD3 and anti-CD28 mAb. EGFR, TGFβ-RII, IRF1 and CK2 mRNA expression 0 min (left) and 15 min (right) after stimulation was analyzed and normalized to EF1α and Teff calculated by delta-delta CT Method. Results are representative for eight independent experiments (n = 8, mean ± SEM, *P < .05; **P < .01). (B) Immunoblot analysis of PAK2 in cytoplasmic (left) and nuclear (right) fractions of human Teff and Treg. Cells were unstimulated (0h) or stimulated with anti-CD3 and anti-CD28 mAbs for 24h or 48h. Blotting with actin (left) and laminin (right) antibodies as loading control. One representative experiment out of three is presented. (C) RT-PCR for PAK2 in Teff was performed after stimulation with anti-CD3 and anti-CD28 mAb at 1h and 16h upon activation (n = 3, mean ± SEM, *P < .05).

In the next step, expression of significantly regulated kinases (Table 1) was analyzed on mRNA or protein level. We were able to confirm increased levels of TGF-β-RII and EGFR mRNA early after Treg activation compared to activated Teff (Fig 4A). Additionally, IRF-1, a factor known to be activated through EGFR-STAT1-STAT3 pathway, was also induced after activation. Interestingly, whereas CK2 kinase activity in kinome array was significantly upregulated comparing activated Treg to Teff (Fig 3), mRNA levels of this kinase showed no significant differences. As depicted for PAK2, protein expression was only found in the cytoplasmic fraction of activated Teff starting 24h after activation with a maximum expression at 48h but not in Treg (Fig 4B). On mRNA level, PAK2 expression in Teff was present 1h upon activation (Fig 4C), suggesting that small amounts of PAK2 protein is expressed already early after activation, not efficiently detectable by western blot. These results obtained by real time PCR and western blot analysis resembled only in part those obtained by kinome profiling, demonstrating the urgent need of differentiation between kinase expression levels and kinase activity. Nevertheless, further proof for differential regulation of PAK2 comes from other phosphorylated PAK2 substrates on the array. However, whereas differences were seen, these were not significant in kinome array and thus were not investigated in more detail in the present study.

**Fig 5:**
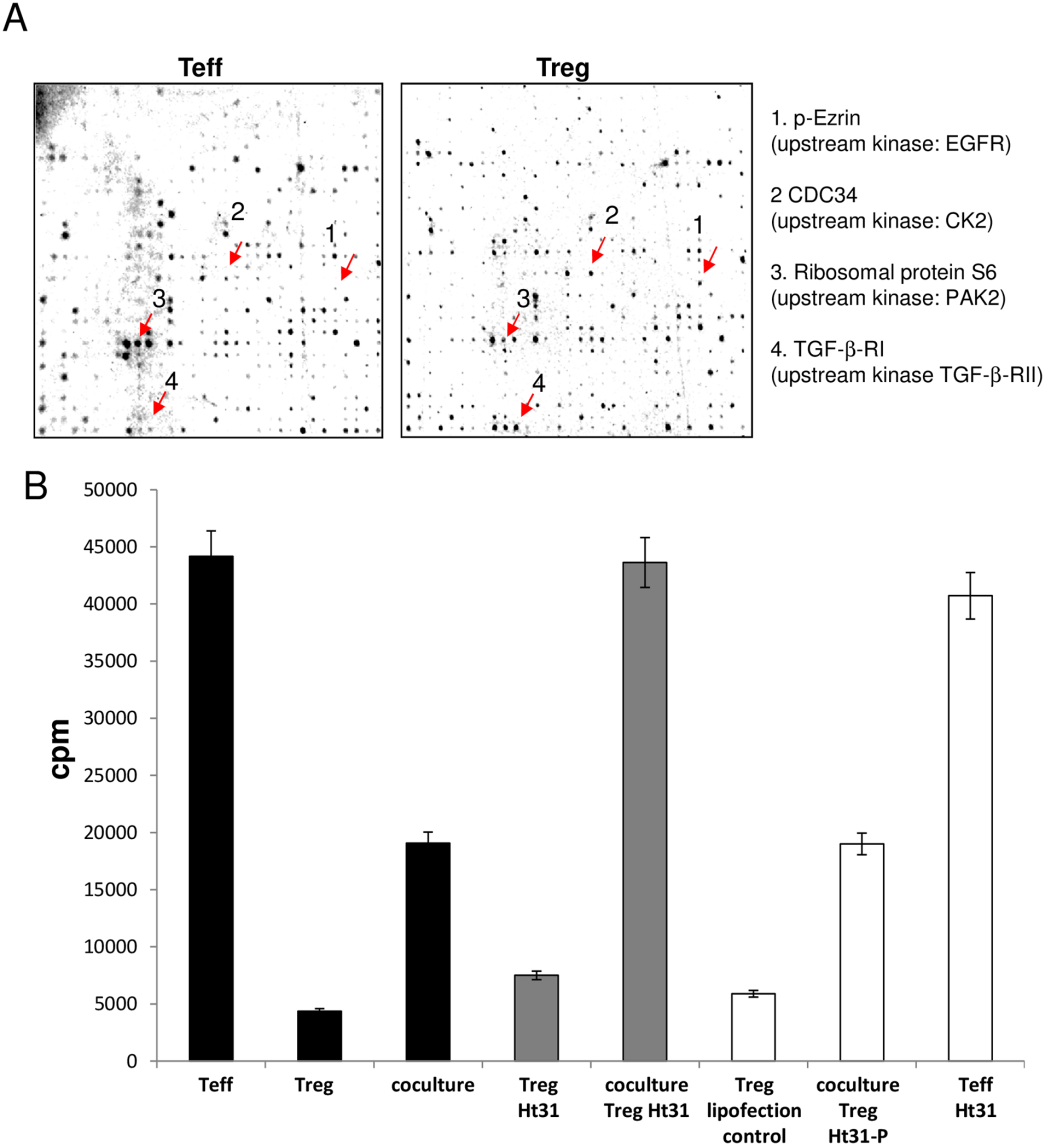
EGFR/Ezrin complex is important for Treg function. (A) Activated Teff (left panel) and Treg (right panel) were subjected to kinome array as described. Scans of the peptide arrays after incubation with lysates of activated Teff and Treg in the presence of [γ-33P]ATP. Each spot represents phosphorylation of a specific substrate through kinase activity as present in the different lysates. Notice the specific patterns for Treg and Teff. (B) Treg were treated with either Ht31, a control peptide (Ht31P) or left untreated as indicated and were cocultured with Teff. As an additional control, lipofected Treg as well as Ht31 treated Teff were used (n = 3).

As an example for functional analysis we chose the Ezrin/EGFR complex that showed increased activity in Treg compared to Teff. One of the target substrates phosphorylated by EGFR and also identified in the present kinome array is the cytoskeleton-associated protein Ezrin, belonging to the A-kinase anchoring proteins (AKAPs, Fig 5A). From cancer biology it is known that EGFR and Ezrin form a functional complex, however, their exact interactions are still elusive [29,30]. As kinome data revealed a significant phosphorylation of the substrate Ezrin in activated Treg due to upregulated activity of its upstream kinase EGFR, we next investigated the impact of EGFR/Ezrin on Treg function. First, we established the functional importance of Ezrin using the anchoring inhibitor peptide Ht31. After incubation of Treg with Ht31, they were co-cultured with Teff and their suppressive activity was assessed. As shown in Fig 5B, suppressive capacity of Ht31-treated Treg was completely abrogated. These results give a first hint that the functional complex of EGFR/Ezrin revealed by kinome profiling is of importance for Treg function in humans and should be analyzed in more detail.

**Fig 6:**
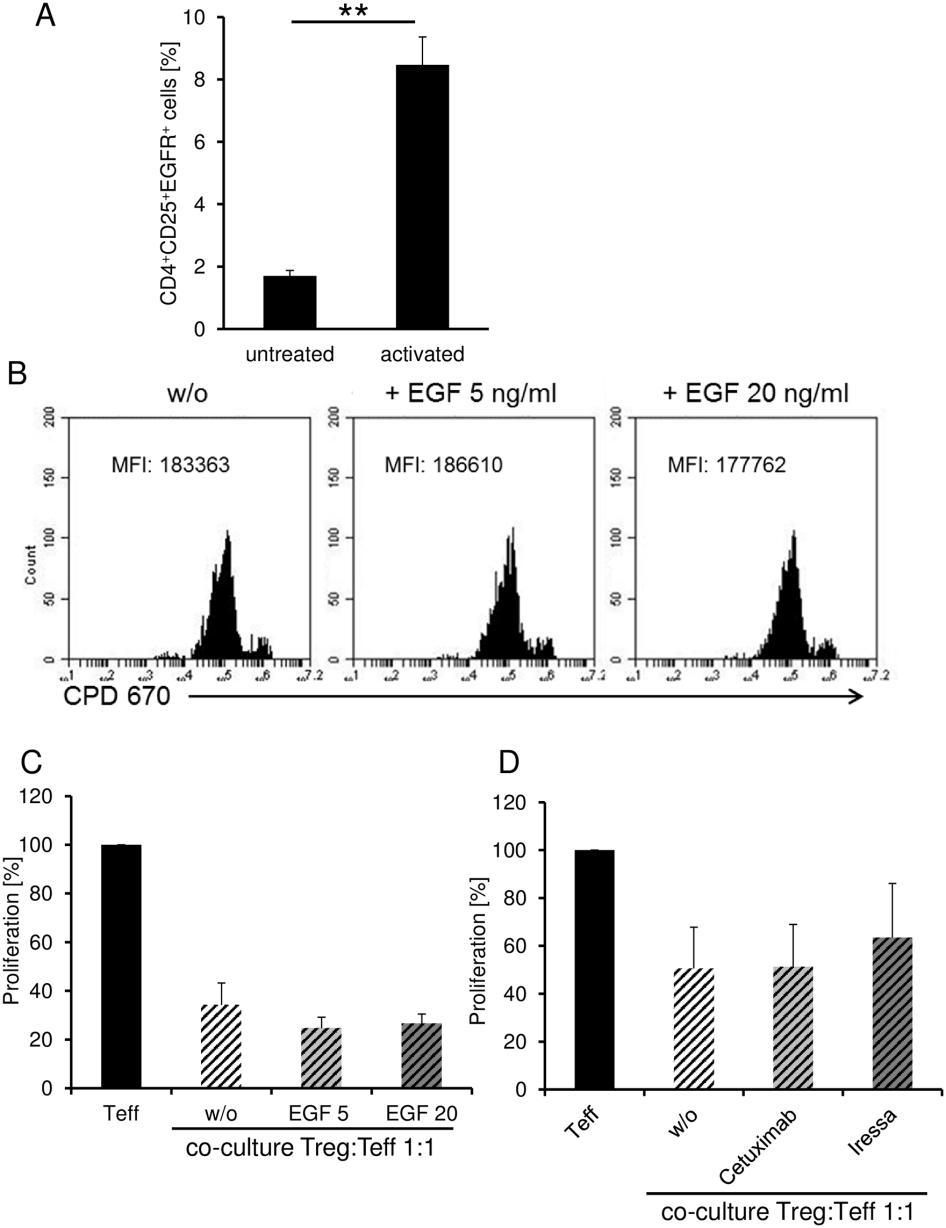
Role of EGFR for Treg function. (A) CD4^+^CD25^+^ Treg were isolated and stimulated with anti-CD3 and anti-CD28 mAb. Cells were stained with biotinylated EGF to detect EGFR expression on cell surface. Diagram show pooled data of three independent experiments (mean ± SEM, **P < .01). (B) CPD670-labeld CD4^+^CD25^+^ Treg were isolated and stimulated with anti-CD3 (0.5μg/ml) mAb and CD3-depleted PBMC in presence or absence of EGF (5 or 20ng/ml). Proliferation was determined on day 4. Histogram shows a typical result of three independent experiments. (C) CFSE-labeled CD4^+^ Teff and CD4^+^CD25^+^ Treg were stimulated with anti-CD3 (0.5μg/ml) mAb and CD3-depleted PBMC in presence or absence of EGF (5 or 20ng/ml). Proliferation was determined on day 4. Data are normalized to Teff proliferation alone. Results show the pooled data of 3 independent experiments (mean ± SEM). (D) Treg were pre-cultured with or without Cetuximab (1μg/ml) or Iressa (0.5μM) for 1h as indicated and then cocultured with Teff. Data are normalized to Teff proliferation alone. Results show the pooled data of 4 independent experiments (mean ± SEM).

Beside the analysis of Ezrin as a differentially phosphorylated substrate detected in kinome array, we furthermore investigated the functional role of its corresponding kinase EGFR in more detail. Early after activation, EGFR was upregulated on the surface of a small subset of Treg (Fig 6A). However, stimulation of Treg with EGF did not change their anergic phenotype (Fig 6B) nor their suppressive capacity significantly (Fig 6C). Additionally, blockade of EGFR signaling *in vitro* showed also no significant effect on suppressive activity of Treg (Fig 6D). Treatment of Treg with the EGFR kinase inhibitor Gefitinib (Iressa) mildly however not significantly inhibited Teff suppression in coculture experiments (Fig 6D). Whereas the functional relevance of increased CK2 levels in activated murine Treg for the suppression of TH2-mediated allergic immune responses in the lung could recently be demonstrated *in vivo* [11], other candidate kinases such as EGFR have to be analyzed in the same way in appropriate disease models *in vivo* e.g. in tumors.

## Discussion

Regulatory T cells have an important impact on the immune homeostasis. After TCR engagement, Treg acquire a typical phenotype including expression of Foxp3, up-regulation of GARP, anergy, low cytokine production and suppressive properties. It is probable that also the regulation of protein kinases and thus distinct signaling pathways are important for Treg phenotype and function.

Traditional genetic and biochemical approaches can certainly provide information about functional marker molecules in immunoregulatory cells. Nevertheless, there is typically pursued one gene or protein or pathway at a time. Thus, to get a real insight into the complex regulatory networks of human immune cells, a more comprehensive approach is needed in order to reveal active and relevant signaling pathways. Towards this end, techniques using peptide arrays have begun to be applied successfully in a variety of cell populations [25,31,32] generating descriptions of possible distinct signaling pathways in distinct cells. As not only quantification of kinase activities but also comparisons between different conditions can easily be performed, the outcoming results help to identify important marker molecules. It should be noted that the limited specificity of protein kinases towards their peptide substrates on microarrays needs data validation using other independent methods, such as PCR, western blot or flow cytometric analysis. Nevertheless, this kinome profiling method uniquely allows for the detection of non-canonical, if not unknown, kinase pathways, as is the case with the EGFR-Ezrin connection in this paper.

Over the last years, array technologies have enabled the determination of the proteome and genome as well as of the kinome in human immune cells that helped to elucidate molecular mechanisms to gain insights into cell function. In more detail, arrays are able to define those factors that participate in signaling pathways and thus control cell fate and function. Knowing which signaling pathways are being used in immune cells, especially regulatory T cells are of critical importance. Konig et al. [33] analyzed kinases relevant for human Treg phenotype. Nevertheless, in contrast to our approach, *ex vivo* expanded instead of freshly isolated Treg were investigated. This approach holds the danger of changes of Treg phenotype and function during culture and thus is not unrestrained comparable to the *in vivo* Treg situation. Recent investigations have shown that upon *in vitro* expansion, human Treg down-regulate cell signature genes, such as FOXP3, CTLA4, ICOS, IKZF2 and LRRC32/GARP, but up-regulate a set of T helper lineage-associated genes, especially T helper type 2 (Th2)-associated, such as GATA3, GFI1 and IL13 [34]. Furthermore, Konig et al. used mass spectrometry and thus revealed protein mass rather than functional activity of Treg kinases. Nevertheless, in agreement to the study of Konig et al. we also found that the majority of kinases were not exclusively expressed in either of both populations, underlining the validity of our findings. Moreover, our own comparative kinome profiling with mouse Treg and Teff cells showed identical results to a very large degree [11]. Thus, kinome profiling investigating functional active kinases through phosphorylation of corresponding substrates for human Treg at different stages of activation in comparison to Teff as performed in our study is an important approach for identification of novel markers and gene regulation and thus helps us to gain a more detailed insight into Treg biology.

In the present study, a total of 192 active kinases were analyzed in resting and activated regulatory and effector T cells using the “kinome profiling technique”. To the best of our knowledge this is the first time that this array has been applied to analyze human Treg *ex vivo*. We detected a panel of kinases that was differentially regulated in Treg compared with Teff, thus allowing the identification of a number of pathways and biological functions overrepresented in Treg (e.g. CK2, EphB2 or EGFR). On the other hand, we also found kinases with a reduced activity in Treg such as PAK2.

PAK2 belongs to the PAK I family group. It is a serin/threonine kinase that binds to and is stimulated by the activated forms of the small GTPases, Cdc42 and Rac. It is involved in the regulation of cellular processes such as gene transcription, cell morphology and motility as well as apoptosis [35]. PAK2 has been shown to be cleaved by caspase 3 during apoptosis. This cleavage activates PAK2 by releasing its kinase domain which contributes to morphological changes during apoptosis.

Among the upregulated kinases in Treg, EGFR is known to be critically involved in tissue development and homeostasis but also in the pathogenesis of cancer which resulted in a widespread use of EGFR-targeted treatment in cancer patients in the past. In cancer, EGFR has been shown to have growth inhibitory effects as it induced the expression of IRF-1 via phosphorylation of STAT1 and STAT3 [36]. This study shows that cells acquiring sustained high activity of EGFR are able to activate a module of IFN-associated genes including IRF-1 through activation of STAT1 and STAT3. IRF-1 mediates growth arrest *in vitro* and could be responsible for the inhibition of cell proliferation.

So far, increased EGFR expression has not been discussed in the context of human Treg function. Recently, Zaiss et al. [37] have shown in mice that murine Foxp3^+^ Treg express EGFR under inflammatory conditions. Yuan et al. reported that Amphiregulin activated murine Treg through the EGFR pathway in a murine model of hepatocellular carminoma [38]. These results revealed EGFR as a component in regulation of local immune responses in different diseases.

In the context of the discussed literature, our findings of elevated expression in activated human Treg and the functional relevance of the EGFR/Ezrin complex define EGFR as an important key molecule for local immune responses. Stimulation of EGFR induces a complex cascade of phosphorylation and activation events and thus can determine T cell fate and function. It has been shown that Ezrin-Radixin-Moesin can form a complex with EGFR which regulates subcellular localization and thus affects the activity of intracellular signaling pathways activated after EGF ligand binding [39,40][39,40]. Thus targeting of this immune regulatory mechanism may additionally contribute to the therapeutic successes of EGFR-targeting treatments in cancer patients. Nevertheless, we found no significant modulation of Treg suppressive capacitiy in our *in vitro* system using EGFR stimulatory or inhibitory agents. Probably, significant effects could not be observed because only a small population of peripheral Treg showed surface expression of EGFR. In addition, other Treg characteristics beside suppressive capacity should be analyzed in future studies. However, Yuan et al. described that in a murine model of hepatocellular carcinoma intratumoral Treg expressed significant higher levels of functional EGFR than peripheral Treg. *In vivo*, modulation of murine Treg function through EGFR signaling was demonstrated after Amphiregulin stimulation [38]. Accordingly, future studies have to analyze the relevance of EGFR for human Treg activation in appropriate *in vivo* systems. In the present study, we also detected phosphorylation of GAB1 or VAV2 in Treg following activation of EGFR. Of note, we did not find significant differences between Treg and Teff for these substrates in kinome array.

Our results indicate that also Ezrin, belonging to the AKAPs, has an important impact on T regulatory function. Interestingly, Ezrin interacts with PKA, a kinase also more active in activated human Treg compared to Teff. Thus, in future studies, it will be of great interest to investigate in more detail the role of Ezrin-dependent PKA signaling since increased intracellular cAMP levels and PKA-dependent CREB phosphorylation are essential for Treg function [39].

Another kinase that was identified by a preceding kinome array in murine T cells that turned out to be significantly increased in activated Treg was CK2. Based on these data in a recent work by Ulges et al. [11], we demonstrated the importance of the activity of protein kinase CK2 for function of a distinct Treg subset with significant relevance in allergic immune responses in a murine model. Importantly, in the actual study investigating human Treg, CK2, as well as EGFR, showed a significant difference in activity between human Treg and Teff in kinome array. Nevertheless, mRNA level did not show different expression levels. This underlines the importance of combining different and independent assays for functional testing of Treg marker molecules and their relevance for Treg phenotype. Our data demonstrate the significant difference between the presence of kinases and their functional activity.

In summary, our analysis identified a panel of previously unrecognized Treg molecules contributing to an extended human Treg signature that helps to specify key elements of their regulatory properties and to grow the knowledge on Treg subsets and function. This study combined for the first time cellular kinome array technique along with bioinformatical analysis and functional immunological assays in order to validate the obtained results. However, extended analysis and validation of those proteins and their contribution to Treg activity and function has to be performed in future studies. Importantly, we have to keep in mind, that phosphorylation of substrates in this array in some cases is only an indirect hint on the different regulation of the upstream kinase as many signaling pathways and thus kinases have overlapping substrates. This underlines the importance of independent validation of array results combining different assays/methods as well as functional testing of Treg marker molecules and their relevance for Treg phenotype. In addition, in the presented work we focused on the differential regulation of activated Treg versus activated Teff early after stimulation. Nevertheless, there is still a huge amount of data that has to be interpreted from different points of view. One possible analysis could be the comparison of distinct Treg subsets or the differences from resting versus activated Treg at different time points after activation in order to study their concrete signaling cascade after TCR stimulation. In conclusion, the detailed investigation of kinomes that control the functional properties of human Treg but also human Teff opens the possibility to identify new molecular targets and pathways for the development of effective immunotherapies against unwanted T cell activities in allergy, autoimmunity and cancer.

## Supporting Information

S1 Fig Isolation of human T cell populations. (A) Treg and Teff were isolated from leukapheresis products of healthy donors as described before using magnetic bead isolation. (B) The resulting cell populations were either activated using stimulation with anti-CD3 and anti-CD28 mAb or left untreated. All four populations were subjected subsequently to kinome profiling as described.
(TIF)

S2 Fig Technique for kinome profiling. (A) For kinome array samples, Treg and Teff were isolated and stimulated as described. Cells were lysed and the peptide array mix was added onto the chip. (B) A dedicated image analysis procedure was used for quality control and quantification of spot intensities as described in Material and Methods. Each kinase substrate was located by optimally aligning the grid of spotted substrates (upper part). Spot intensities were corrected for local background intensity. Following 2log-transformation, net spot intensities were subsequently analyzed. Data from three technical replicates were exported to an excel sheet for further analysis. Control spots on the array were analyzed for validation of spot intensities between the different samples (lower part). Furthermore, inconsistent data (e.g. spots with irregular shape or position) were excluded from further analysis. Slides were normalized by median-centering and a t-test was used to detect significantly different phosphorylation levels between different conditions.
(TIF)

S3 Fig Kinetic profiling of significantly different regulated kinases. Graphical illustration of the kinetic profiles of the 9 additional molecules differentially regulated in Treg (blue) versus Teff (red). For every time point measured expression of the peptide for all three samples is depicted for both cell types as dots. The curves depict the mean expression for the peptides, separated for Treg and Teff, over all time points (x-axis: time, y-axis: peptide mass).
(TIF)
