# Supplementary figures and images for: Kinome Profiling of Regulatory T Cells: A Closer Look into a Complex Intracellular Network
Source: PLoS One. 2016 Feb 16;11(2):e0149193. doi: 10.1371/journal.pone.0149193 (PMC4755507; doi:10.1371/journal.pone.0149193)

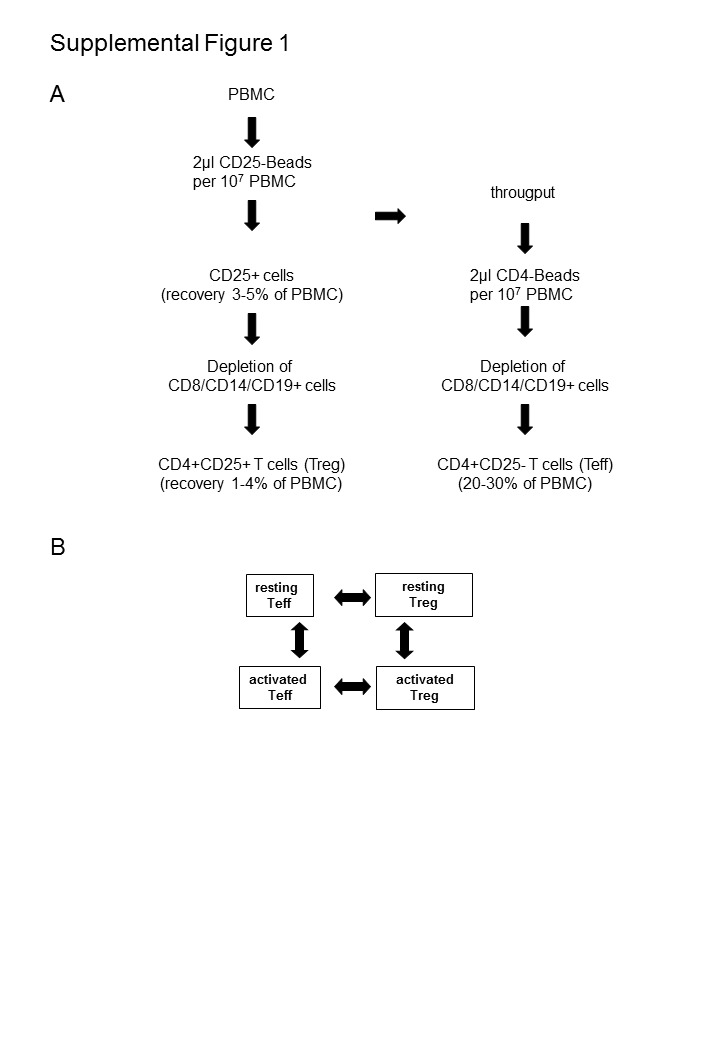

Supplement: S1 Fig — (A) Treg and Teff were isolated from leukapheresis products of healthy donors as described before using magnetic bead isolation. (B) The resulting cell populations were either activated using stimulation with anti-CD3 and anti-CD28 mAb or left untreated. All four populations were subjected subsequently to kinome profiling as described. (TIF) [file pone.0149193.s001.TIF]

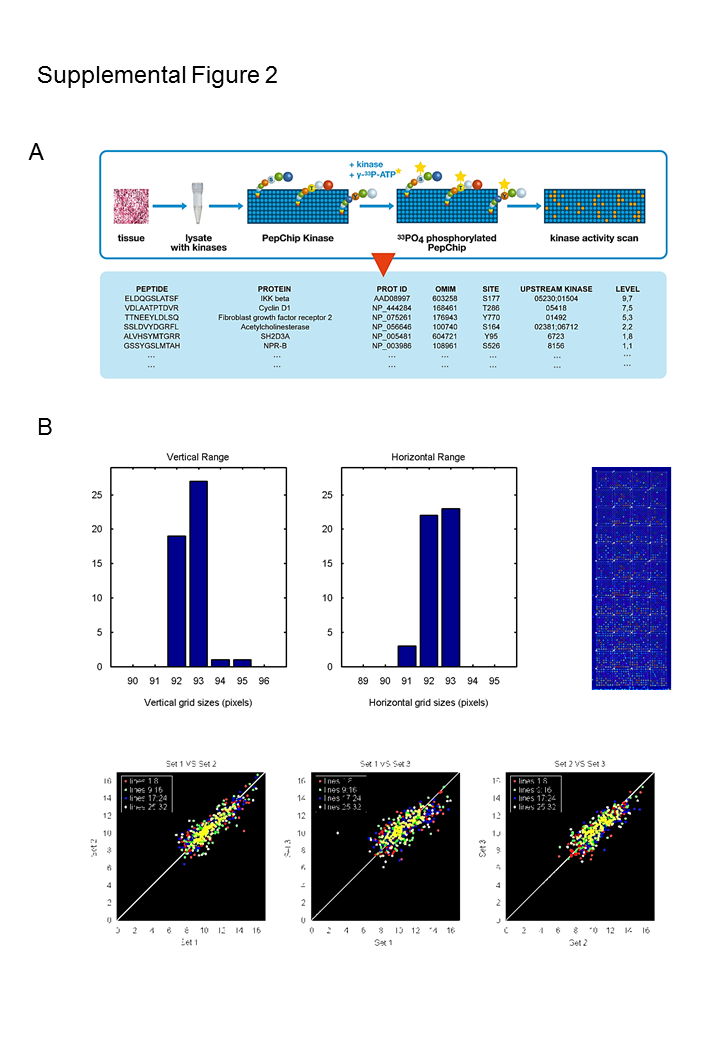

Supplement: S2 Fig — (A) For kinome array samples, Treg and Teff were isolated and stimulated as described. Cells were lysed and the peptide array mix was added onto the chip. (B) A dedicated image analysis procedure was used for quality control and quantification of spot intensities as described in Material and Methods. Each kinase substrate was located by optimally aligning the grid of spotted substrates (upper part). Spot intensities were corrected for local background intensity. Following 2log-transformation, net spot intensities were subsequently analyzed. Data from three technical replicates were exported to an excel sheet for further analysis. Control spots on the array were analyzed for validation of spot intensities between the different samples (lower part). Furthermore, inconsistent data (e.g. spots with irregular shape or position) were excluded from further analysis. Slides were normalized by median-centering and a t-test was used to detect significantly different phosphorylation levels between different conditions. (TIF) [file pone.0149193.s002.TIF]

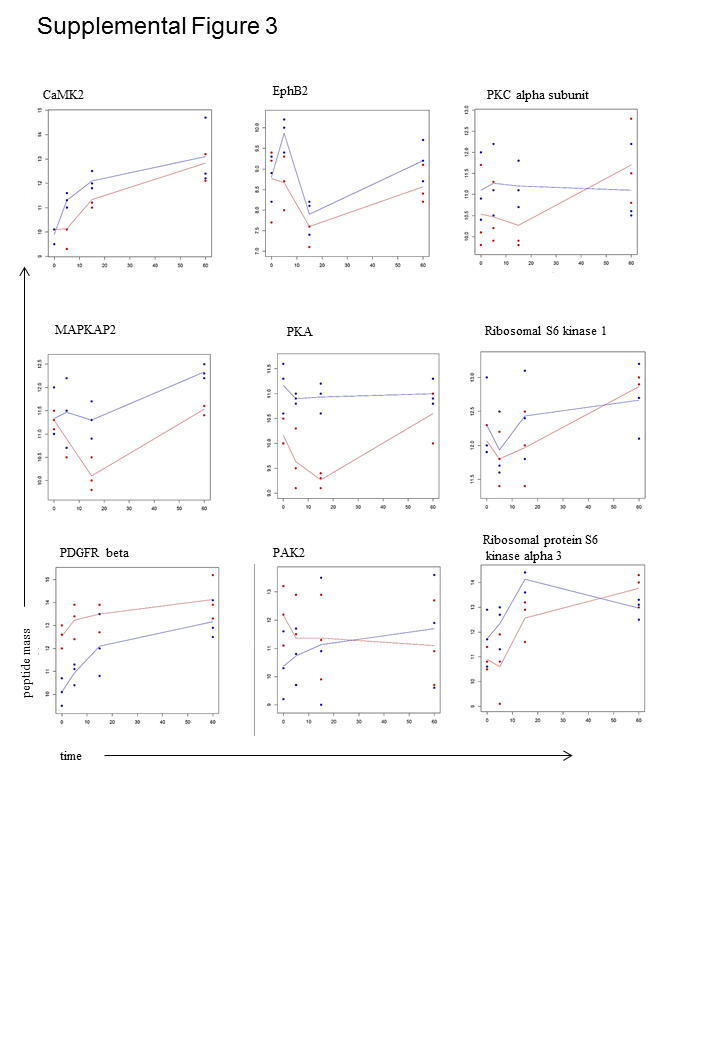

Supplement: S3 Fig — Graphical illustration of the kinetic profiles of the 9 additional molecules differentially regulated in Treg (blue) versus Teff (red). For every time point measured expression of the peptide for all three samples is depicted for both cell types as dots. The curves depict the mean expression for the peptides, separated for Treg and Teff, over all time points (x-axis: time, y-axis: peptide mass). (TIF) [file pone.0149193.s003.TIF]
